# Simultaneous Water‐Specific and Fat‐Specific T1 and Fat Fraction Mapping of the Abdomen in Free Breathing Conditions With a Radially Encoded MP2RAGE

**DOI:** 10.1002/nbm.70297

**Published:** 2026-05-08

**Authors:** Nadège Corbin, François Maingault, Aurélien J. Trotier, Emile Kadalie, Laurence Dallet, Marc Biran, Sylvain Miraux, Eric Thiaudière, William Lefrançois, Emeline J. Ribot

**Affiliations:** ^1^ Univ. Bordeaux, CNRS, CRMSB, UMR 5536, F‐33000 Bordeaux, France Bordeaux France; ^2^ Physical Sciences Research Platform Sunnybrook Research Institute Toronto Ontario Canada; ^3^ Biomedical Imaging Platform pIBIO, UAR3767, CNRS Bordeaux France

**Keywords:** free breathing, MP2RAGE, PDFF, radial, water‐specific T1

## Abstract

The purpose of this study is to enable 3D abdominal imaging and quantitative parameter mapping in free breathing conditions and simultaneously provide proton‐density fat fraction (PDFF), water‐specific and fat‐specific T1 maps. A radially encoded MP2RAGE with alternated fat and water selective pulses was implemented and validated on a phantom containing gadolinium (Gd) and pork fat at different concentrations. Comparison with MR spectroscopy and imaging techniques of reference was performed in vitro. Multiple experiments were carried out on healthy volunteers and individuals with a record of liver disease and benign bone marrow lesions to evaluate the method's repeatability, accuracy and potential for clinical application. In vitro water or fat specific T1 was in agreement with estimates provided by reference methods over a wide range of mixture ratios. PDFF was strongly correlated with spectroscopy (Pearson coefficient of 0.98) and other imaging techniques although underestimated due to the imperfect pulse selectivity profile. Images free of ghosting artefacts were acquired in vivo on seven volunteers. The whole abdomen was imaged, as well as a large part of the spine (from T11 to L5). Parametric maps provided repeatable estimates in the liver, the bone marrow and the subcutaneous fat that were consistent with values reported in literature and other imaging techniques. The acquisition time could be halved without significantly affecting the quantitative values. Overall, high‐contrast MP2RAGE abdominal images, water‐ and fat‐ specific T1 maps and PDFF maps were achieved in a single 3D acquisition under free breathing.

## Graphical abstract

A radially encoded MP2RAGE with alternated fat and water selective pulses to enable 3D abdominal imaging and quantitative parameter mapping in free breathing conditions and simultaneously provide proton‐density fat fraction, water‐specific and fat‐specific T1 maps. The isotropic resolution and the large coverage enable the estimation of the quantitative parameters in various organs and pathologies.

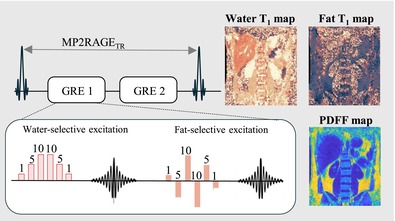

**Abbreviations**
BMI: body mass index
CSE: chemical shift encoding
ETL: echo train length
FOV: field of view
GRE: gradient recalled echo
IQR: interquartile range
LUT: look‐up table
MP2RAGE: Magnetization Prepared 2 Rapid Acquisition Gradient Echoes
PBS: phosphate buffer saline
PDFF: proton density fat fraction
RF: radiofrequency
ROI: region of interest
SNR: signal‐to‐noise ratio
STEAM: stimulated echo acquisition mode
SWALI: simultaneous water‐fat separation and T1 mapping of the whole liver
TE: echo time
TI: inversion time
TR: repetition time
UNI: UNIFORM image
VFA: variable flip angle

## 1 Introduction

Quantitative MRI significantly aids in diagnosing and monitoring many pathologies. The longitudinal relaxation time T1 is particularly interesting for abdominal imaging to assess the severity of cholangitis [1], diagnose fatty liver disease [2, 3] and chronic pancreatitis [4], or differentiate lower and higher grade renal cell carcinoma [5]. However, the presence of infiltrated fat within the abdominal organs largely decreases the estimated T1. Instead, the water‐specific T1 can also be very informative when diagnosing liver pathologies [6, 7, 8]. Also, fat‐specific T1 helps differentiate between different subtypes of fat, providing insights on damages related to obesity for instance [9].

In addition to T1, the proton density fat fraction (PDFF) is also a biomarker for numerous abdominal pathologies. PDFF can reliably diagnose and grade hepatic steatosis [10] or even predict and evaluate the risk for liver disease. PDFF has also been considered as a promising biomarker for pancreatic cancer [11] and a useful tool to differentiate benign and malignant vertebral lesions [12] or to assess bone fragility [13]. Only few studies, all relying on the chemical‐shift encoding (CSE) approach [14, 15], proposed a joint estimation of the component‐specific T1 and PDFF [16, 17, 18, 19] in the abdomen. Two of them have limited coverage and spatial resolution because they are limited to one breath hold [17, 18].

Indeed, a significant challenge in abdominal imaging stems from both respiratory and gastrointestinal motion. Breath holds during acquisitions help reduce motion induced artefacts but can be restrictive for paediatric, elderly or severely ill patients and may lead to misregistrations between scans acquired across multiple breath‐holds. An alternative is to synchronize the acquisition with the subject's respiration. However, motion can still occur during the acquisition window, scan time increases and irregular breathing becomes an issue [20].

To extend the coverage or the spatial resolution without imposing long breath holds or relying on respiratory gating, technical development is required. An interesting solution is to sample the k‐space radially in order to acquire the centre multiple times, making it more robust to motion [21]. This is the approach adopted for SWALI [16] for instance.

Whereas SWALI relies on multiple inversion times, the focus here is on the Magnetization Prepared 2 Rapid Acquisition Gradient Echoes (MP2RAGE) sequence, a T1 mapping technique already well‐established in neuroimaging [22]. In addition to providing high T1‐weighted contrast images necessary for reliable segmentation, this efficient method enables the estimation of 3D T1 maps by combining only two images. This combination eliminates T2* and receive field contribution, and an optimal choice of sequence parameters makes the approach robust to the transmit field efficiency. This study aims to optimize the MP2RAGE sequence for abdominal application, leveraging its established advantages. Our focus is on two fundamental aspects:

1. Maintaining patient comfort while supporting large coverage and high spatial isotropic resolution: 3D radial sampling is employed to mitigate motion sensitivity under free breathing,
2. Joint estimation of component‐specific T1 and PDFF: Binomial RF pulses are implemented to alternatively excite fat and water components to obtain multiple parametric maps in a single scan.

In this study, the accuracy of the abdominal MP2RAGE sequence is evaluated both on phantom and in vivo. Test–retest acquisitions are performed on healthy volunteers to investigate the repeatability of the method. The versatility of the proposed sequence is further evidenced by the estimation of the quantitative parameters in various organs and pathologies.

## 2 Methods

### 2.1 Principle

#### 2.1.1 MR Pulse Sequence

The standard Cartesian encoding was replaced by a 3D Kooshball golden‐angle radial sampling scheme [21]. This pattern enables isotropic resolution and a large coverage while allowing free breathing and ensuring motion robustness. The 3D golden‐means radial trajectory [21] guarantees a pseudo‐random filling of the k‐space.

Successive water‐specific spokes were rotated by the golden angle, and fat‐specific spokes were rotated 90° relative to the preceding water‐specific spokes. During one MP2RAGE_TR_ (duration separating two successive inversion pulses), the radial spokes of both GRE trains were identical. At the *n*th MP2RAGE_TR_, the spokes were acquired as the successors of those previously acquired at the (*n*−1)th MP2RAGE_TR_.

**FIGURE 1:**
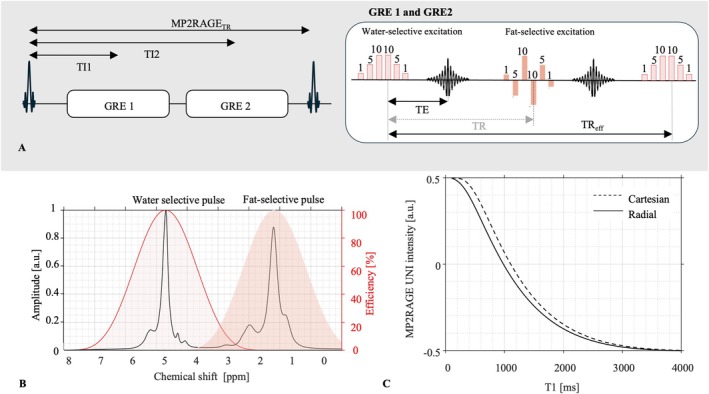
(A) Sequence diagram of the modified MP2RAGE sequence. (B) Water and fat spectrum obtained from a mixture of pork fat, water and agar (black) with the NMR spectroscopy protocol (STEAM) overlayed on water‐selective (shaded area with solid line) and fat‐selective (shaded area without line) excitation profiles of the binomial pulses. (C) Look‐up table (LUT) of the radial MP2RAGE protocol of this study (solid line), overlayed on the LUT corresponding to a cartesian protocol with identical timing parameters (dashed line).

Binomial excitation pulses composed of six hard subpulses allowed water‐ or fat‐only acquisitions, called water‐ and fat‐specific hereafter (Figure 1A,B). For water, the coefficients [1−5−10−10−5−1] were applied to get an excitation efficiency of more than 90% over a range of 112 Hz around the water and minimal excitation efficiency (<0.1%) over a range of 207 Hz around 1.3 ppm. Similarly, selective excitation of fat was performed using the coefficients [$1-\bar{5}-10-\bar{10}-5-\bar{1}$], where the overbars denote a 180° phase shifted pulse. Each subpulse had a duration of 60 μs, with an equidistant duration of 1.15 ms between two consecutive pulse centres resulting in a total duration pulse of 5.81 ms. This relatively long pulse prevented the excitation of fat components like α‐olefinic (2 ppm), α‐carboxyl (2.2 ppm) with the water‐selective pulse while mitigating the excitation of the water component by the fat‐selective pulse in regions suffering from B0 inhomogeneity (Figures 1B and S1).

Water and fat protons were alternately excited during one train, resulting in a twice longer TR for each component (*TR*_*eff*_ = 2*TR) and an effective echo train length (*ETL*_*Eff*_) of ETL/2.

#### 2.1.2 Reconstruction Process

First, water‐ and fat‐specific GRE1 and GRE2 images, S_1_^*Water*^, S_1_^*Fat*^, S_2_^*Water*^ and S_2_^*Fat*^ were reconstructed only from odd or even spokes, respectively. A nonuniform Fourier transform with density compensation function was applied using the BART toolbox [23]. The coil sensitivities were estimated using only the GRE2 spokes of the water‐specific images. Iterative reconstruction with parallel imaging and compressed sensing regularization was applied using the ‘pics’ function of the BART toolbox. Spatial regularization with wavelet transform was applied with regularization parameter of 0.01. The two GRE images per selective excitation were then combined to generate water‐ and fat‐specific MP2RAGE UNI images, using the standard equation described in [22].

#### 2.1.3 Calculation of Water‐ and Fat‐Specific T1 Maps

The Bloch equations used to build the look‐up table (LUT) were adapted from Marques et al. [22] to compute the water‐ and fat‐specific T1 maps (Figure 1C; Appendix A). The signal of the centre points of all spokes were averaged together, which differs from the Cartesian sequence and therefore modifies the signal modelling at the inversion times [24]. These new parameters shifted the LUT curve towards shorter T1 compared to Cartesian encoding (Figure 1C), leading to a measurable range of T1 from 100 to 3500 ms. The inversion efficiency was set to 0.9, based on empirical measurements in one participant's liver (Figure S5) and consistent with the range used in brain applications [22, 25]. Effective flip angle correction was also performed to account for B0 inhomogeneity. Although this correction was applied on the presented data, the correction had negligible impact with this protocol due to the small flip angles (details in Figures S3 and S4). Component‐specific T1 (*T*1_*Water*_ and *T*1_*Fat*_) was estimated by corresponding MP2RAGE UNI values and the associated LUT.

#### 2.1.4 Determination of the Fat Fraction

According to the equation of the MP2RAGE signal derived in the appendix of Marques et al. [22], the signal of GRE1 ($S_{1}^{\text{Comp}}$) and GRE2 ($S_{2}^{\text{Comp}}$) of any of the two components (*Comp =* Fat or *Comp =* Water) can be reduced to

(1) $$S_{1}^{\text{Comp}}=S_{0}^{\text{Comp}}f_{1}\left(T1_{\text{Comp}}\right)$$

and

(2) $$S_{2}^{\text{Comp}}=S_{0}^{\text{Comp}}f_{2}\left(T1_{\text{Comp}}\right)$$

With *f*_1_ and *f*_2_ known functions only depending on sequence parameters (Equations A3 and A4), *T*1_*Comp*_ the T1 of water or fat (estimated above) and $S_{0}^{\text{Comp}}=B_{1}^{-}e^{-\mathrm{TE}/T2_{\text{Comp}}^{*}}M_{0}^{\text{Comp}}$ the apparent proton density depending on the B_1_^−^ received field, *M*_0_^*Comp*^ the initial magnetization and *T*2*_Comp_ the transverse relaxation time.

For robustness, this system of two equations with one unknown (either $S_{0}^{\mathrm{Fat}}$ or $S_{0}^{\text{Water}}$) was solved with the ordinary least square estimator for each of the component.

$S_{0}^{\mathrm{Fat}}$ and $S_{0}^{\text{Water}}$ were then combined as follows:

(3) $$S_{0}^{\mathrm{Fat}}/\left(S_{0}^{\mathrm{Fat}}+S_{0}^{\text{Water}}\right)=\frac{e^{-\mathrm{TE}/T2_{\mathrm{Fat}}^{*}}M_{0}^{\mathrm{Fat}}}{e^{-\mathrm{TE}/T2_{\mathrm{Fat}}^{*}}M_{0}^{\mathrm{Fat}}+e^{-\mathrm{TE}/T2_{\text{Water}}^{*}}M_{0}^{\text{Water}}}$$

and assuming T2* equivalent for the two components [6, 19], PDFF in percent was derived:

(4) $$\text{PDFF}=100*S_{0}^{\mathrm{Fat}}/\left(S_{0}^{\mathrm{Fat}}+S_{0}^{\text{Water}}\right)=100*M_{0}^{\mathrm{Fat}}/\left(M_{0}^{\mathrm{Fat}}+M_{0}^{\text{Water}}\right)$$

### 2.2 Evaluation

The experiments were performed on a Siemens Prisma 3T MRI (XR gradients: 80 mT/m maximum amplitude, 200 T/m/s maximum slew rate) using the whole‐body transmit coil and an 18‐channel body surface coil combined with 18 elements of the table‐mounted spine array for reception.

Key parameters of the abdominal MP2RAGE protocol are in Table 1. To estimate the effective flip angle in the presence of B0 inhomogeneity affecting the binomial pulse (Figure S1), B1^+^ and B0 field maps were acquired (Table 1). This enables the generation of a voxel‐specific LUT, incorporating the transmit field efficiency and off‐resonance frequency at that location.

**TABLE 1:** Sequence parameters.

	Abdominal 3D radial MP2RAGE	Single voxel STEAM with multiple inversion times	3D spoiled GRE sequence (VIBE) with variable flip angles	Single voxel STEAM with multiple echo times	CSE approach with 3D multi echo GRE	B1 + mapping with SS‐preturbo flash	B0 mapping multiecho GRE
Water‐specific T1	✔	✔				For T1 mapping correction (VFA and MP2RAGE)	
Fat‐specific T1	✔	✔	✔				
PDFF	✔			✔	✔		
TR	10.1 ms	10 s	15 ms	10 s	13 ms	8420 ms	220 ms
TE	3.45 ms	30 ms	2.54 ms (nonselective) 2.88 ms (water‐selective)	7 TEs from 20 to 110 ms	6 TEs equally spaced from 1.09 ms to 7.38 ms	1.83 ms	5.19 and 7.65 ms
Acquisition matrix	256 × 256 × 256		288 × 210 × 10 (in vitro) and 96 × 72 × 10 (in vivo)		160 × 103 × 30	64 × 64 × 23	64 × 56 × 20
Voxel size	1.4 × 1.4 × 1.4 mm^3^	8 × 8 × 35 mm^3^	1.23 × 1.23 × 4 mm^3^ (in vitro) and 3.6 × 3.6 × 4 mm3 (in vivo)	8 × 8 × 35 mm^3^	2.3 × 2.4 × 3 mm^3^	6 × 6 × 8 mm^3^	5.5 × 5.5 × 8 mm^3^
Bandwidth	1260 Hz/pixel	2000 Hz	890 Hz/pixel	4000 Hz	1560 Hz/pixel	490 Hz/pixel	260 Hz/pixel
Flip angle	4° for both GRE trains	90°	3,8,13 and 18°	90°	4°	8°	60°
Additional parameters	TI = 668 ms and 2800 ms MP2RAG*E*_*TR*_ = 5 s *TR*_*Eff*_ = 20.2 ms 9216 radial spokes per component GRE train length of 64 per component	TM = 15 ms averages 25 TI from 50 to 2450 ms	Two acquisitions with and without water selective option (normal mode)	TM = 15 ms 4 averages			Two acquisitions: one with the shim volume copied from the VFA protocol, the other from the MP2RAGE protocol
In vitro	12 min	17 min: 36 s	2 × 55 s	5 min: 34 s	16 s	17 s	2 × 21 s
In vivo		N/A	2 × 23 s	N/A			

#### 2.2.1 Phantom Experiment

A container for multiple sample vials (MultiSample120, Gold Standard Phantoms, United Kingdom) comprised 14 tubes and was filled with tap water surrounding the tubes. Four tubes were made out of 1.5% agarose diluted in Phosphate Buffer Saline (PBS) mixed with varying concentrations of Gadolinium (DOTAREM, Guerbet, France): 0, 0.02, 0.1 and 0.3 mM. Another tube composed of only pork fat was included (lard).

A solution of 1.5% agarose diluted in PBS and 0.02 mM of Gadolinium (DOTAREM, Guerbet, France) was prepared. Nine tubes were prepared as a mixture of this solution and pork fat at different volume concentrations (from 10% to 90%).

In addition to the maps computed from the abdominal MP2RAGE protocol, component‐specific T1 and PDFF maps were obtained from standard methods for comparison:

- Component‐specific T1 in each vial was estimated from MR spectroscopy with a set of single voxel STEAM protocols with inversion pulses (STEAM_T1_). Nonselective and water‐selective T1 maps were also obtained from the vendor variable flip angle (VFA) approach. Key parameters are available in Table 1. The VFA protocol was applied twice, once with water selection relying on a binomial pulse with three subpulses (maximum available) and once without water‐selection.
- PDFF in each vial was estimated from MR spectroscopy with a set of single‐voxel STEAM sequences with different echo times (STEAM_T2_). PDFF maps were also obtained from CSE approaches from a 3D multiecho GRE sequence to maximize SNR while maintaining reasonable spatial resolution in the three directions. Key parameters are available in Table 1.

The protocols, with the exception of spectroscopy, were specifically designed to fit in one breath hold and be used subsequently in vivo. Nevertheless, the spatial resolution had to be increased for the VFA acquisitions in vitro because of the small size of the vials.

#### 2.2.2 In Vivo Experiment

A total of seven volunteers (three males and four females, between 29 and 51 years old) were scanned with the approval of the local ethics committee and the informed written consent of the participants. Five participants were considered as healthy volunteers, as they had no record of liver disease. One volunteer, V4, showed a high record of ferritin in the liver (287 μg/L 1 month before the acquisition) and another one, V7, suffered from hepatic steatosis. The volunteers were asked to lay down with their arms inside the body coil and to breath normally for the abdominal MP2RAGE protocol. Breath holds in expiration were required for the comparative methods detailed thereafter.

Three datasets were collected, one including multiple acquisition techniques (Dataset 1), a second one evaluating the repeatability of the proposed sequence (Dataset 2) and a third one including one volunteer with hepatic steatosis (Dataset 3). Demographics of the volunteers are available in Table 2.

**TABLE 2:** Details of the volunteers included in the three datasets.

	Sex	Age (years)	Weight (kg)	Height (cm)	BMI (kg/m^2^)	Medical record	Datasets
V1	F	43	48	165	17	/	1 & 2
V2	M	31	80	190	22	/	1
V3	F	38	58	165	21	/	1 & 2
V4	F	52	80	170	27	High record of ferritin in the liver (287 μg/L 1 month before the acquisition)	1 & 2
V5	F	30	68	167	24	/	2
V6	M	30	95	180	29	/	2
V7	M	46	82	168	29	Hepatic steatosis	3

##### 2.2.2.1 In Vivo Dataset 1

Three healthy volunteers and V4 were scanned with the abdominal MP2RAGE protocol under free breathing conditions and with the VFA protocol and the CSE acquisition under breath holds. B1+ and B0 field mapping protocols also required breath holds.

##### 2.2.2.2 In Vivo Dataset 2

Test/retest experiments were performed on five volunteers (including V4) to evaluate the repeatability of the abdominal MP2RAGE. The volunteers came out of the scanner between the two acquisitions.

##### 2.2.2.3 In Vivo Dataset 3

The volunteer suffering from hepatic steatosis (V7) was only scanned once with the abdominal MP2RAGE protocol.

#### 2.2.3 Data Processing of Comparative Methods

##### 2.2.3.1 Spectroscopy Data Processing

Data acquired with both STEAM protocols were processed with the Suspect library (github.com/openmrslab/suspect), including coil combination, 0th‐order and 1st‐order phase correction. Water and methylene peaks amplitude across TI (or TEs) were fitted to Equation (5) (or Equation 6) to estimate component‐specific T1 (or T2, respectively).

(5) $$\begin{array}{c} y=M0^{\text{Comp}}*\left(1-\left(1-\cos\left(\mathrm{eff}*\pi \right)\right)*\exp\left(-\frac{\mathrm{TI}}{T1_{\text{Comp}}}\right)\right) \end{array}$$

with *eff* = 0.9 to be consistent with the abdominal MP2RAGE processing.

(6) $$y=M0^{\text{Comp}}*\exp\left(-\mathrm{TE}/T2_{\text{Comp}}\right))$$

The spectral fitting of the fat and water contents was performed with LCModel [26] using the model ‘lipid‐8’ on the acquisition with the shortest TE (TE_1_). The PDFF was computed as follows:

(7) $$\mathrm{PDF}F_{\text{STEAM}}=100*\frac{S_{\mathrm{Fat}}e^{\frac{TE_{1}}{T2_{\mathrm{Fat}}}}}{S_{\mathrm{Fat}}e^{\frac{TE_{1}}{T2_{\mathrm{Fat}}}}+S_{\text{Water}}e^{\frac{TE_{1}}{T2_{\text{Water}}}}}$$

with $S_{\mathrm{Fat}}$ being the sum of the eight components estimated by the fitting of the first echo. Given the poor reliability of the T2 estimates in tubes with low concentrations of one of the components, the same values of T2, that is, 50 ms for water and 30 ms for fat (estimated from the tube with equal concentration of fat and water), were used for all the tubes. In the tube with only pork fat, the residual water was not corrected for T2 decay, as no gadolinium was added.

##### 2.2.3.2 VFA Data Processing

T1 estimation from the VFA acquisitions was performed with the QMRLab library (https://qmrlab.org/) after in‐house correction of the effective flip angle altered by the interaction between B0 inhomogeneity and the selective pulses (see Figure S2).

##### 2.2.3.3 CSE Data Processing

CSE acquisitions were processed with the multispectral fitting algorithm [27] and the 2012 ISMRM challenge model, implemented in the open‐source CREAM library [28].

#### 2.2.4 Accuracy and Repeatability Assessment

##### 2.2.4.1 Phantom Dataset

Mean and average estimates across the regions of interest (ROIs) located in each tube were computed for each acquisition technique.

##### 2.2.4.2 In Vivo Dataset 1

ROIs in the liver and the bone marrow in a slice common to all the protocols were manually drawn. The mean and the standard deviation across the ROIs of all the estimated parameters were compared across techniques.

##### 2.2.4.3 In Vivo Dataset 2

The two abdominal MP2RAGE acquisitions were coregistered after estimation of the rigid transformation based on the GRE2 image with SPM12 [29] (https://www.fil.ion.ucl.ac.uk/spm/), a widely used open‐source software package for imaging data analysis. Circular ROIs in the liver (10 pixels radius), the bone marrow (5 pixels radius) of the second lumbar vertebrae and the subcutaneous fat at the hip level (10 pixels radius) were manually drawn on the first acquisition. Mean and standard deviation within the ROIs were computed for each acquisition. The repeatability of the measurements was assessed by means of Bland–Altman plots representing the absolute T1 and PDFF differences, with respect to their corresponding average. The 95% confidence interval was computed by calculating the limits of agreement (mean difference ± 1.96 * standard deviation).

To highlight the benefit of a method providing high spatial isotropic resolution and large coverage, the component‐specific T1 and the PDFF of the bone marrow across the vertebras was measured on this dataset. Seven circular ROIs (4 pixels radius) were manually placed on each vertebra between T11 and L5. Mean and standard deviations within the ROIs were computed for each of the estimated parameters. A linear mixed model effect with random effect for intercept was fitted to the data to infer on a linear increase of the parameters along the spine. The *p* value associated to the slope term was computed and deemed significant if *p* < 0.001.

##### 2.2.4.4 In Vivo Dataset 3

Mean and standard deviation of component‐specific T1 and PDFF were computed in one ROI manually drawn within the liver.

#### 2.2.5 Acceleration Capability Assessment

Data from one of the volunteers (V2) underwent retrospective undersampling, corresponding to 10‐, 8‐, 6‐, 4‐ and 2‐min acquisition duration to assess the potential for acceleration. The undersampling consisted of discarding all the radial spokes acquired during the *N* last MP2RAGE_TR_, with N∈{24,48,72,96,120}, given that a golden angle sampling approach was used. The voxel‐wise normalized difference of the PDFF and the water‐specific T1 in the whole volume was analysed with respect to the original PDFF and water‐specific T1. Voxels in the background were excluded. Interquartile range (IQR) of the normalized difference and the median were computed.

## 3 Results

### 3.1 Phantom Experiments

**FIGURE 2:**
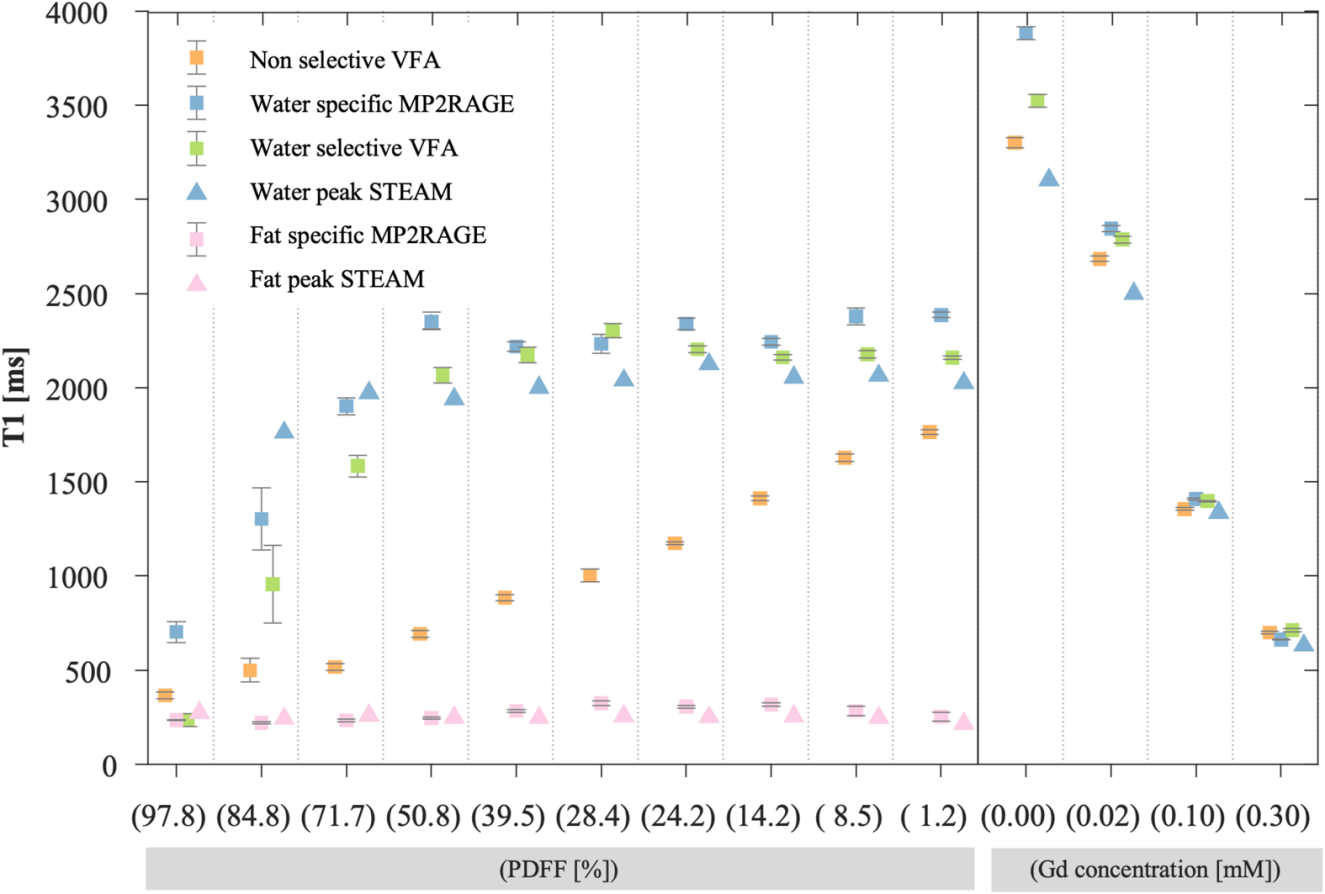
In vitro experiment: T1 estimates. Fat‐ (pink) and water‐specific (blue) T1 values obtained from the modified MP2RAGE sequence (squares) and the spectroscopy experiments (triangles). T1 estimates obtained from the variable flip angle approach, with (green squares) and without (orange squares) water selection are also reported. Vials are identified by their concentration in gadolinium for the fat free vials and by their estimated PDFF with the spectroscopy protocol for the others.

The water‐specific T1 maps showed T1 values close to spectroscopy‐based estimates with differences lower than 5.4% for T1 less than 2 s (Figure 2). However, 25% and 12% higher values were measured with the abdominal MP2RAGE sequence than with spectroscopy for vials without gadolinium and with the lowest concentration (0.02 mM) of Gd, respectively. The difference between the abdominal MP2RAGE and the water‐selective VFA did not exceed 10.2% for all the vials. This was also the case with the nonselective VFA except for the tube without Gd where the difference reached 17.1%.

The fat‐specific T1 was similar to the one estimated by spectroscopy, with a maximum difference of 67 ms (Figure 2). The fat specific T1 obtained from spectroscopy was stable across a wide range of fat contents (249 ± 15 ms). However, we observed slightly higher T1 values (from 280 to 325 ms) with the abdominal MP2RAGE sequence when the fat content was between 10% and 50% and smaller T1 values (between 220 and 245 ms) for fat fraction above 50%.

As expected, the nonspecific T1 obtained with the VFA decreased with the increase of PDFF. As observed with spectroscopy, the water‐specific T1 of the VFA and the abdominal MP2RAGE were stable for the tubes with fat content smaller than 70%, ranging from 2218 to 2387 ms for the MP2RAGE and from 2066 ms to 2300 ms for the water‐selective VFA. A small decrease of the water‐specific T1 was measured with spectroscopy for the vial with 84.8% of fat (1762 ms). In parallel, the water‐specific T1 decreased to 1300 ms with the abdominal MP2RAGE, and even to 950 ms with the VFA protocol, suggesting a failure of the water selectivity.

**FIGURE 3:**
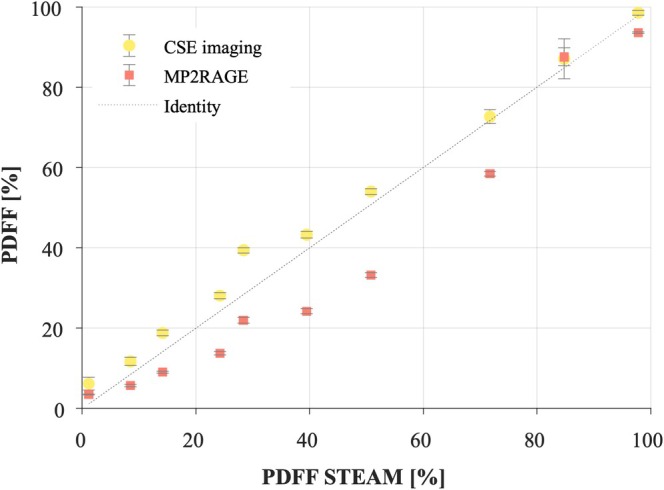
In vitro experiments: PDFF estimates. Estimated PDFF with the modified MP2RAGE approach (red squares) and the 3D CSE approach (yellow circles). The estimated PDFF are shown with respect to the PDFF estimated with the spectroscopy protocol.

Abdominal MP2RAGE‐based PDFF estimates showed a strong correlation with spectroscopy and CSE imaging as shown by the Pearson correlation coefficients (0.98 for both) (Figure 3). The correlation coefficient of the MP2RAGE‐based PDFF and the CSE‐based estimate was 0.96. Nevertheless, PDFF measured with the MP2RAGE protocol underestimated the fat fraction compared to CSE and spectroscopy measurements (maximized for the tube with 51% fat), which rely on multispectral fitting of the fat components. It must be noted that the tube with estimated PDFF of 85% turned out to be very inhomogeneous (large standard deviations). This comes from difficulties in preparing such mixture.

### 3.2 Water‐ and Fat‐Specific T1 in the Abdomen

**FIGURE 4:**
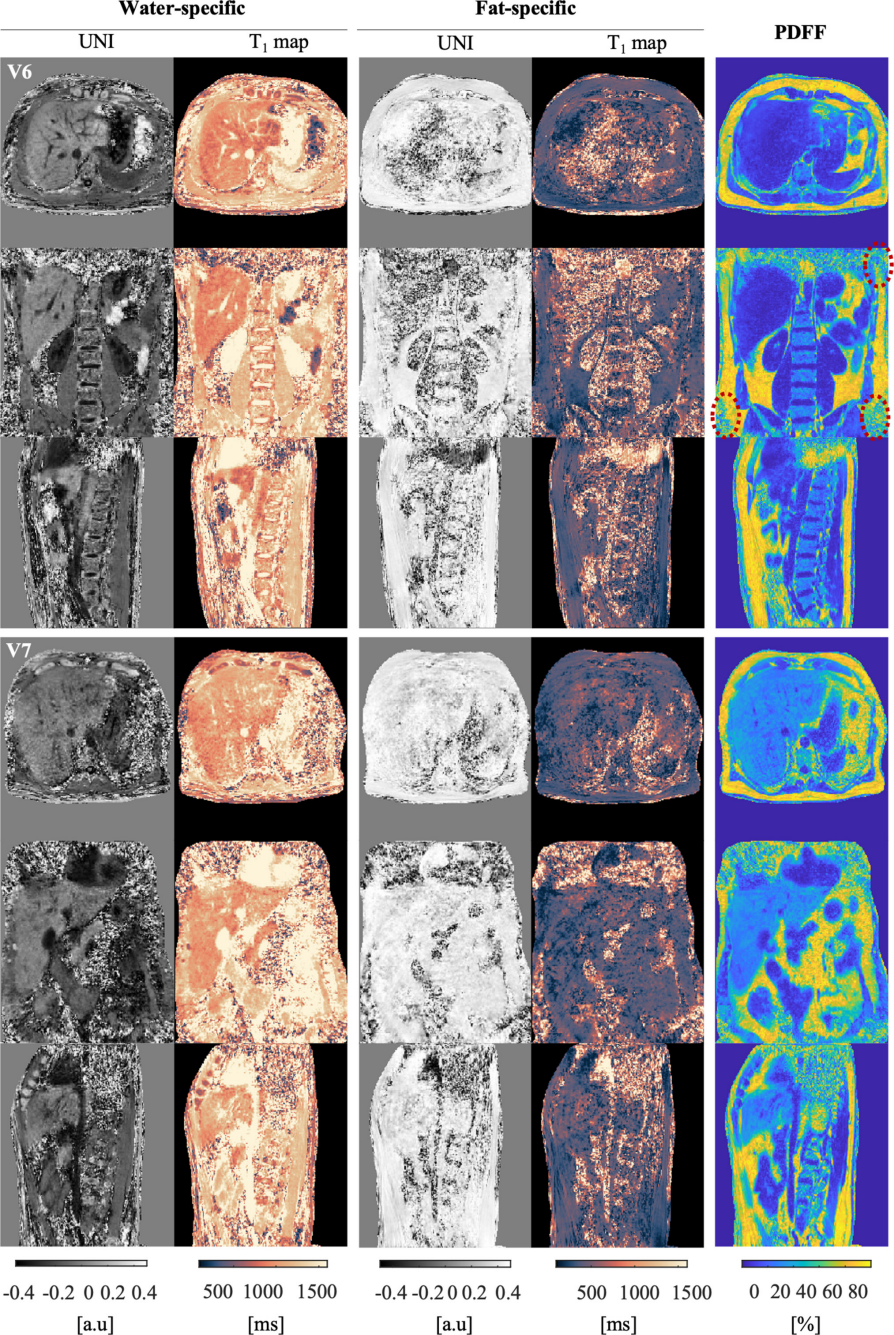
Abdominal MP2RAGE images of V6 (top) and V7 (bottom) and the corresponding T1 and PDFF maps calculated with the water‐ and fat‐specific data. The three orientations are shown to appreciate the large FOV of measurement. Red dashed circles emphasize regions of poor SNR attributed to low receiver coil sensitivity. V7 was suffering from hepatic steatosis.

MP2RAGE UNI images, component‐specific T1 and PDFF maps are shown in Figure 4 for two volunteers. Despite acquisitions carried out during free breathing, no ghosting artefact related to the respiratory motion was visible, and this was the case for all the volunteers.

Water‐specific UNI images provided high contrast. Hepatic blood vessels, intervertebral discs and their internal structures (nucleus pulposus and annulus fibrosus) were particularly well delineated. No chemical shift artefact could be observed due to the absence of fat signal. Subcutaneous and visceral fat tissue appeared as salt‐and‐pepper noise.

Conversely, only subcutaneous and visceral fat were visible on the fat‐specific UNI images. In this case, water‐only containing tissue appeared with a salt‐and‐pepper background.

The protocol enabled to cover a large FOV, from the liver to the sacrum. However, we did not observe obvious deformations that could be attributed to gradient nonlinearity. Nevertheless, noisy regions in the lower and upper side regions, corresponding to the border of the receive coils, were observed in all maps (Figure 4, red circles). In some volunteers, high B0 inhomogeneity was also observed close to the lungs, resulting in a swap of the fat and water signal (see Figures S3 and S4).

#### 3.2.1 Comparison With Standard Methods (Dataset 1)

**FIGURE 5:**
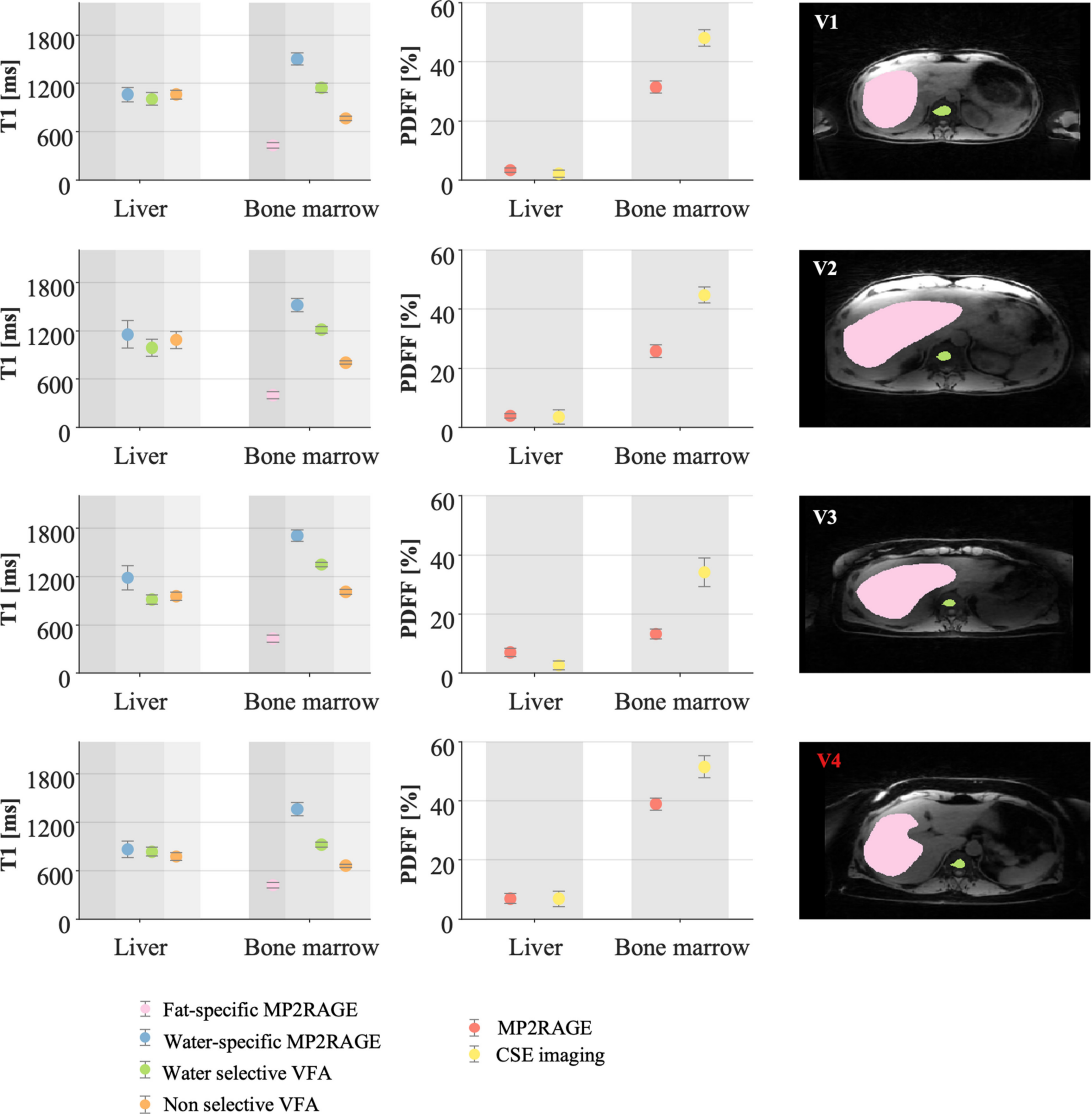
In vivo acquisitions, comparison across techniques. Specific water and fat T1 and PDFF estimated in the liver and the bone marrow in three healthy volunteers and one volunteer with high ferritin record (V4). PDFF was estimated with the modified MP2RAGE method and the 3D CSE imaging method. Water‐specific T1 was estimated with the radial MP2RAGE and the water‐selective VFA protocol. Additionally, fat‐specific T1 estimated with the radial MP2RAGE and the T1 estimated with the nonselective VFA protocol are reported. ROIs for each volunteer are shown on the right (liver in pink and bone marrow in green).

In line with phantom experiments, when the fat content was negligible, water‐specific T1 obtained from VFA or abdominal MP2RAGE were similar (Figure 5), as observed in the liver of healthy participants (1133 ± 63 ms for the MP2RAGE and 970 ± 48 ms for the VFA, across participants). In the bone marrow, which showed a higher fat content, water‐specific T1 of the abdominal MP2RAGE was always higher (1575 ± 114 ms) than the water‐specific T1 of the VFA (1232 ± 102 ms). As expected, the nonselective T1 of the VFA was shorter (864 ± 130 ms), and the fat‐specific T1 of the abdominal MP2RAGE was even shorter (420 ± 17 ms).

#### 3.2.2 Repeatability (Dataset 2)

**FIGURE 6:**
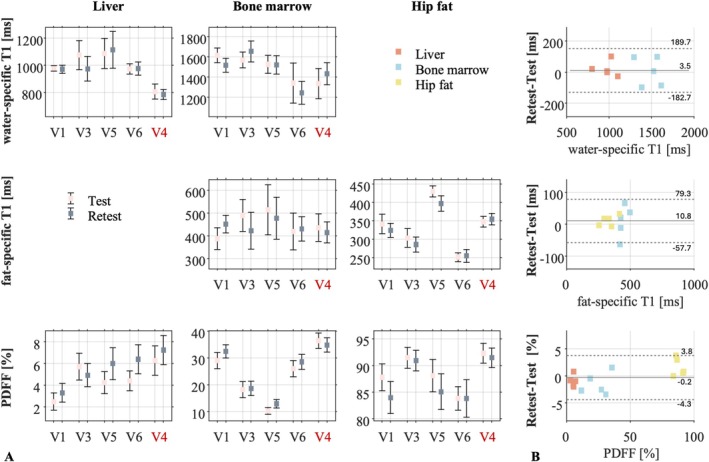
In vivo acquisitions, test–retest experiment. (A) Estimated PDFF, fat‐specific and water‐specific T1 with the abdominal MP2RAGE approach in three ROIs located in the liver, the bone marrow of the first lumbar vertebra and the fat at the hip level. Estimated values for the test and retest experiments are reported. Note that the last volunteer (V4) is the volunteer with high record of ferritin. (B) Bland–Altmann plots of the water‐ and fat‐specific T1 and PDFF in the three ROIs. The dotted lines represent the 95% confidence intervals.

Test–retest repeatability experiments showed that water‐specific T1 differences in the liver were less than 2% except for V3, where the difference reached 6.4% (Figure 6). In the bone marrow, the normalized difference did not exceed 4.9% for all the volunteers.

Fat‐specific T1 differences between the test and retest were < 10.4% in the bone marrow. For the subcutaneous fat, the fat‐specific T1 differences between the test and retest were below 5.3%. The average fat‐specific T1 in the subcutaneous fat was 331 ± 75 ms across healthy participants.

### 3.3 Proton Density Fat Fraction in the Abdomen

#### 3.3.1 Comparison With Standard Methods (Dataset 1)

In line with phantom experiments, the PDFF measured with the CSE method was always higher (PDFF = 42% ± 7%) than the PDFF measured with the abdominal MP2RAGE sequence (PDFF = 23% ± 9%) for nonnegligible fat fractions, that is, in the bone marrow (Figure 5). In the liver, the fat fraction was estimated at 4.7% ± 1.9% across healthy participants with the MP2RAGE and 2.8% ± 0.7% with the CSE approach.

#### 3.3.2 Repeatability (Dataset 2)

The percentage of fat contained in the subcutaneous adipose tissue was quantified between 85.0% and 92.3% (Figure 6), with a test/retest normalized difference of 3% maximum.

In the bone marrow of the first lumbar vertebrae, the test/retest normalized difference of the PDFF was less than 8%, except for V5 where the PDFF was the lowest and the normalized difference reached 16%. However, the absolute difference in the bone marrow did not exceed 3% for all the volunteers.

The normalized difference of the PDFF in the liver was not as low as in the other regions (from 9% to 27%), but the absolute difference did not exceed 2%. Of note, PDFF at the interface of the liver with the lungs was biased in some volunteers, as seen with higher PDFF values of the liver tissue at this location (Figure S4).

### 3.4 Applications

**FIGURE 7:**
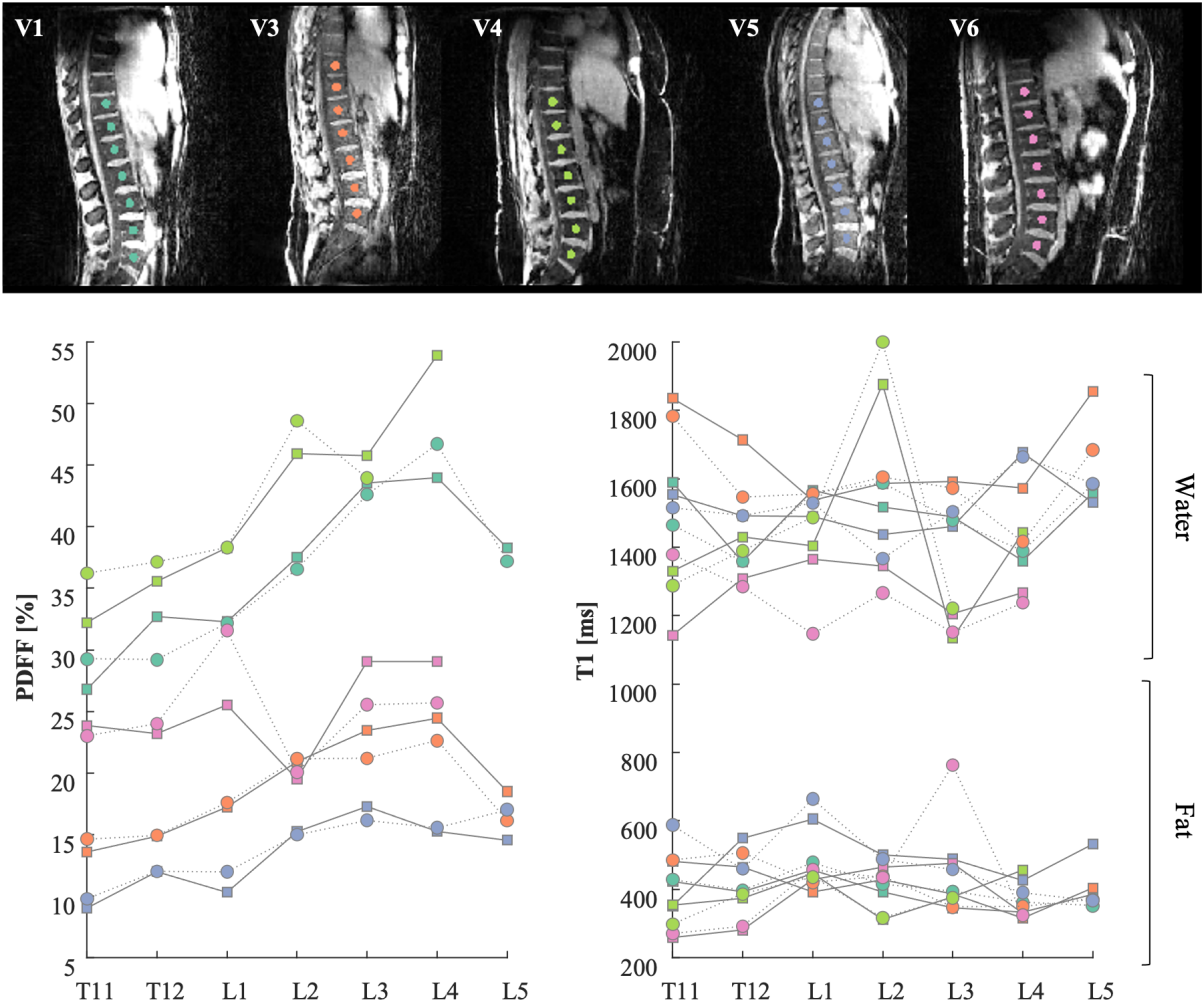
Estimated PDFF and water and fat‐specific T1 of the bone marrow in vertebrae from T11 to L5 for each participant of the test (squares) and retest (circles) experiment. The location of the ROIs is shown in the GRE2 images. Each colour is associated to a volunteer. Data in vertebrae L4 and L5 are missing for V4 and V6 because the field of view of the B1 mapping was not covering this lower part in those participants.

- All the lumbar vertebras were visible in the maps thanks to the large FOV of the sequence (Figure 7). The PDFF of the bone marrow significantly increased (*p* < 1e−10) from T11 to L5, whereas water‐specific T1 and fat‐specific T1 did not show a significant linear variation across the vertebras.
- The second lumbar vertebra of one volunteer (V6) showed a decrease in PDFF (Figure 7). We could also observe an increase of the water‐specific T1 in the secund lumbar vertebrae of V4. Those findings suggest the presence of atypical hemangiomas [30].
- The PDFF of the volunteer with hepatic steatosis disease (Figure 4) was estimated to 27% ± 6% across voxels in the liver, largely above the PDFF measured in the healthy participant livers (4.7% ± 1.9% across participants). In parallel, the water‐specific T1 in the liver was in the range of the water‐specific T1 of the healthy volunteers (1097 ms vs. 1133 ± 63 ms across participants).
- The water‐specific T1 of the volunteer with iron overload (V4) was shorter than the water‐specific T1 observed in the healthy volunteers (Figure 6): 866 ± 203 ms across voxels < 1133 ± 63 ms across participants. The PDFF was in the range of healthy volunteers (6.7% ± 3.3% across voxels vs. 4.8% ± 2.0% across participants).

### 3.5 Acceleration Capability

**FIGURE 8:**
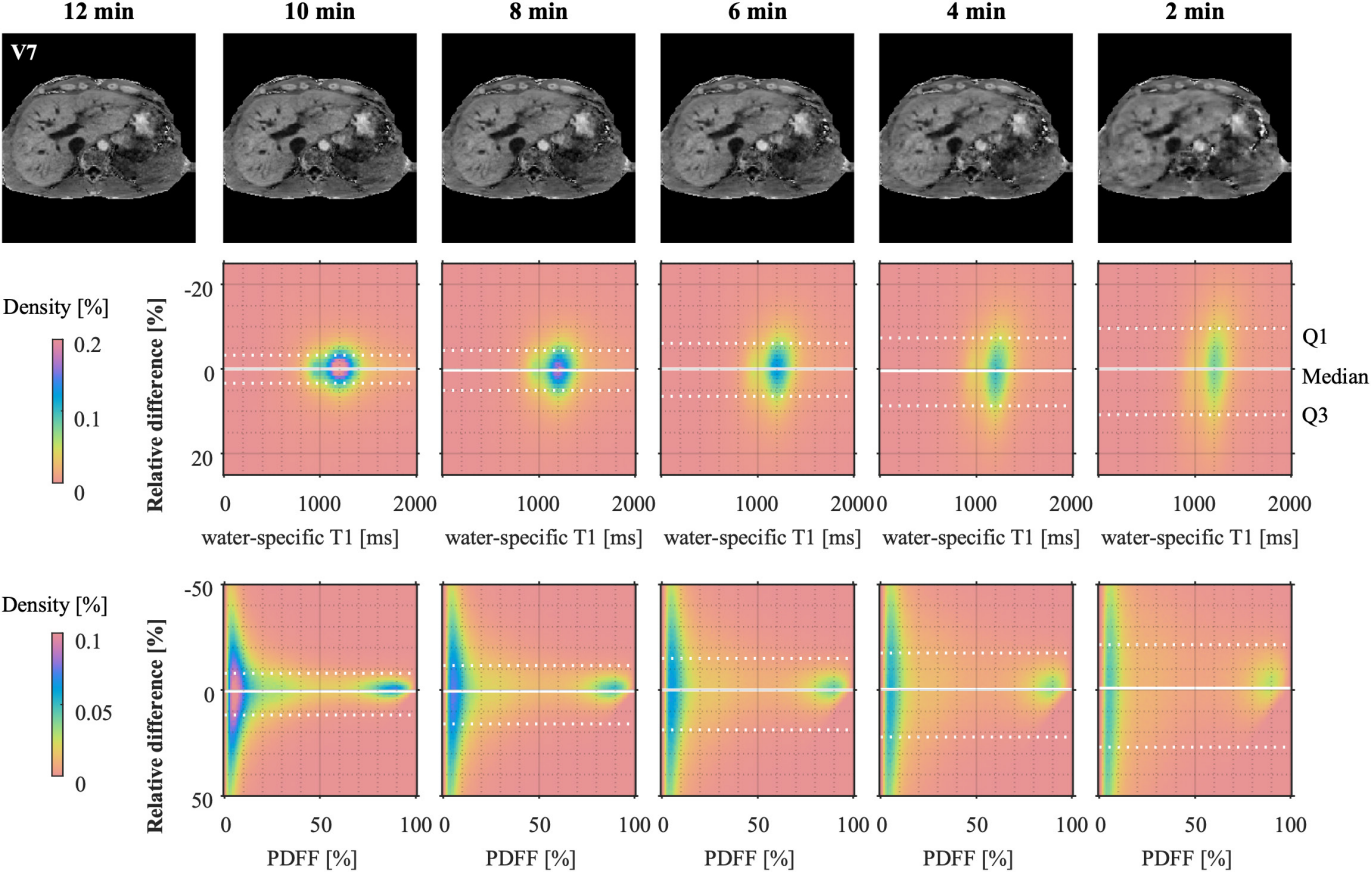
Representative axial slices extracted from a water‐specific MP2RAGE UNI of one volunteer (acquired in 12 min) and calculated after retrospective undersampling with different factors, corresponding to acquisition times of 10, 8, 6, 4 or 2 min (top row). The Bland–Altmann plots below show the voxel‐wise normalized T1 and PDFF differences between the original (12 min) and the undersampled maps in the whole volume (excluding background voxels). Solid white lines show the median. Dotted white lines show the first (Q1) and the third (Q3) quartiles. The colour represents the density of voxels in percentage of the total number of voxels.

Using 6144 or 4608 total spokes resulted in visually comparable weighted images, while reducing the total acquisition time to 8 and 6 min, respectively (Figure 8). Higher acceleration resulted in blurriness, especially around the hepatic vessels.

The mean T1 value remained similar to the initial acquisition for all reconstructions: The mean of the normalized difference did not exceed 0.8% for the water‐selective T1 and the PDFF, even for the highest acceleration factors.

The median of the normalized difference did not exceed 0.5% for water‐specific T1 regardless of the acceleration factor. The IQR of the normalized difference with the original water‐specific T1 in the whole volume was only 9.4% for a simulated acquisition time of 8 min. It increased with the acceleration factor: 12.5%, 16.1% and 20.5% for acquisition times of 6, 4 and 2 min, respectively. The IQR of the normalized difference with the original PDFF was 33.6% for an acquisition time of 6 min, but the IQR of the absolute difference was only 5.4%.

## 4 Discussion

The nongated radially encoded MP2RAGE sequence provides multiple quantitative maps at the abdominal level, without respiratory‐induced ghosting artefacts. This is the first time that the MP2RAGE sequence is used in humans outside of neuroimaging. The use of frequency‐selective excitation pulses enables the reconstruction of both water‐ and fat‐specific images in a single acquisition avoiding the need for subsequent coregistration. To account for the radial sampling and the alternated component selectivity, the derivation of the LUT had to be adjusted.

Water‐specific T1 values are essential to reduce the dependence to fat content, highlighted both in this work, with the nonselective VFA, and in the literature [31, 32, 33]. Among water‐specific T1 mapping methods, many require breath holds and are in 2D [17, 19, 34], which necessitates additional correction to account for the excitation profile.

Among the scarce 3D methods providing access to water‐specific T1, MP‐Dixon‐GRASP [35] only enables water‐specific T1 mapping of the liver with a spatial resolution of 1.37 × 1.37 × 5 mm^3^, in 3 min and 14 s for a limited coverage of 80 mm. Even though this sequence allows free breathing, an external device is needed to record the respiratory traces required for the reconstruction process.

Recently, a 5‐min free breathing 3D multitasking stack‐of‐star multigradient echo sequence was developed to measure water‐specific T1, fat‐specific T1, PDFF and R2* with a similar in‐plane spatial resolution as the current method, but with a 6‐mm partition resolution, and a coverage limited to the liver to minimize the acquisition duration [18]. Very recently, the SWALI method was developed to generate 3D (2.5 mm isotropic) fat‐fraction, water‐specific and fat‐specific T1 maps in 5 min during free‐breathing [16]. Nevertheless, the FOV was smaller (200 mm) than the current method.

Moreover, CSE imaging methods, like SWALI and MP‐DIXON‐GRASP, can be sensitive to T2*. Correcting for T2* requires multiple echoes (at least six) and a longer TR. However, incorporating this into the MP2RAGE GRE train is challenging, as it can compromise T1 sensitivity, effective spatial resolution and acquisition time [36, 37, 38].

The proposed method is thus an interesting alternative to these previously developed methods, as it covers a wide FOV with isotropic resolution and provides component‐specific T1 and PDFF maps without T2* contribution.

### 4.1 T1 Estimation and Comparison With Literature

The acquisitions carried out in vitro revealed a good accuracy in the T1 measurements over the range of abdominal T1 values [39].

Only long T1 values (> 2000 ms) tend to be overestimated by the MP2RAGE protocol compared to spectroscopy. However, VFA measurements also showed discrepancy with spectroscopy for such long T1, probably due to the shorter TR and the imperfect spoiling of the imaging protocols.

Fat‐specific T1 was less than 325 ms regardless of the fat content of the vials. In the pure pork fat vial, a similar T1 value was measured by Hu and Nayak [40] (i.e., 282 ms). Although spectroscopy and abdominal MP2RAGE measurements were comparable, we could observe a small variation of the fat‐specific T1 with the MP2RAGE, which was not present in the spectroscopy measures. As described in the Section 2, the estimation of the fat‐specific T1 component via spectroscopy was limited to the methylene signal (1.3 ppm) to maximize accuracy, whereas the MP2RAGE‐based fat‐specific T1 mainly depends on the methylene, the methyl (0.9 ppm), the β‐carboxyl groups (1.6 ppm) and the ⍺‐olefinic (2.0 ppm). Fortier and Levesque [41] showed that the methylene‐specific T1 did not vary as a function of the fat fraction, but the T1 of the other groups decreased with the fat content. This might explain the small decrease of the fat‐specific T1 measured with the abdominal MP2RAGE with respect to the increase of the fat content.

Similarly, the fatty environment of the water protons leads to a shortening of water‐specific T1 values [40, 41]. This has been corroborated by the current MR spectroscopy results. We also observed a decrease of the water‐specific T1 with the abdominal MP2RAGE and the VFA when the fat content was above 52%, but this is probably confounded by the imperfect pulse selectivity. Indeed, the water‐selective pulses of the abdominal MP2RAGE also excite the olefinic (5.3 ppm, T1 = 421 ms^42^) and the glycerol groups (5.2 ppm, T1 = 413 ms^41^ and 4.3 ppm, T1 = 154 ms^42^) with an efficiency > 85%. The estimated T1 is therefore a combination of the T1 of water and the T1 of these components. The water‐selective pulse of the VFA, which is only a three‐subpulse binomial pulse, also excites the previously mentioned groups, and additionally the α‐olefinic group (2 ppm, T1 = 249 ms [42]) with an efficiency of 12% (as opposed to 0.6% with the abdominal MP2RAGE pulse). This might explain the shorter water‐specific T1 provided by the VFA.

The abdominal MP2RAGE sequence provided component‐specific T1 in various abdominal organs:

- The water‐specific T1 of the liver was 1053 ms ± 76 ms in average across all healthy participants. This is in line with literature data acquired with 2D [17, 34, 43] and 3D sequences [16, 18, 35]. The method was sensitive to the high rate of ferritin present in the liver of V4, also consistent with the literature [44].
- The water‐specific T1 of the bone marrow was approximately twice as long as the ones reported in de Bazelaire [39]. This discrepancy is explained by the use of a nonfrequency‐selective excitation in the latter study. On the contrary, the water‐specific T1 of vertebrae was measured at 701 ± 151 ms [45], 901 ± 13 ms [46] and in the range of 1000–1700 ms [47] at 1.5 T, which corroborates the longer T1 measured at 3 T in the current study (1476 ms ± 133 ms). In parallel, the fat‐specific T1 in the vertebra bone marrow was also found longer (443 ms ± 38 ms) than the ones estimated at 1.5 T in the literature (334 ± 113 ms [45]; 266 ± 2 ms [46] and 260‐320 ms [47]).
- The fat‐specific T1 of subcutaneous fat was in average 329 ± 58 ms. This was in the range of the one measured by de Bazelaire et al. [39] (382 ms) and Leporq et al. [6] (277 ms).
- Although not mentioned in this study, other organs, like the kidneys (well delineated in V6 in Figure 4), can be investigated with this method.

### 4.2 PDFF Estimation and Comparison With Literature

To further explore the potential applications of the current method, PDFF maps were also calculated. The six‐pulse frequency‐selective excitation enables the excitation of the majority of the fat spectrum, without significantly exciting water [48, 49]. In vitro, PDFF could be measured for a wide range of fat mixtures.

The MP2RAGE‐based PDFF were underestimated compared to the CSE imaging method with multispectral fat components fitting approach. With the MP2RAGE approach, the estimated fat content is the weighted sum of the methyl (0.9 ppm), methylene (1.3 ppm), β‐carboxyl (1.6 ppm), α‐olefinic (2 ppm), α‐carboxyl (2.2 ppm) and diacyl‐methylene (2.8 ppm), which is largely dominated by the first four components cited, as they are excited by the binomial pulse with an efficiency > 70%. The other fat components are downweighted, which tends to reduce the estimated global fat content. Furthermore, the olefinic (5.3 ppm) and the glycerol groups (5.2, 4.3 and 4.2 ppm) are excited with an efficiency > 70% by the water‐selective pulse. These fat components are therefore attributed to the water signal, which tends to further underestimate the PDFF.

The repeatability of the PDFF estimates (limit of agreement of the Bland Altmann plot of [−4.3%; 3.8%] calculated over a wide range of PDFF and only a few subjects) was comparable to the repeatability of standard CSE imaging techniques as reported by a meta‐analysis conducted on 9103 measures (limit of agreement of [−2.99%, 2.99%] for PDFF values lower than 40%) [50].

PDFFs were consistent with the ones shown in the literature for the subcutaneous fat [51] and the liver [16, 18]. The sensitivity of the method allowed in this study the detection of an abnormal PDFF in the liver of a patient suffering of hepatic steatosis, whereas the water‐specific T1 was not affected.

Also, the PDFF values can be critical for the differentiation between benign and malignant vertebral lesions [12]. The large FOV of the protocol facilitated the investigation of a long portion of the spine. A large range of PDFF, consistent with the literature, was measured in the bone marrow of the participants of the current study, due to variations in age and gender [12, 52, 53]. The PDFF in the bone marrow was increasing from T11 to L5, in line with the observations of le Ster et al. [45]. However, we did not observe significant decrease of water‐specific T1. This discrepancy might come from the difference in the methods used to separate water and fat components (CSE imaging approach in [45]).

### 4.3 Limitations

The MP2RAGE‐based sequence relies on frequency selective pulses, whose effective flip angle directly depends on the B0 inhomogeneity and the pulse profile. However, the MP2RAGE sequence is known to be robust to B1+ inhomogeneity which makes the component‐specific T1 rather robust to B0 inhomogeneity. For example, with this particular protocol of MP2RAGE, for an off‐resonance frequency of 200 Hz, the excitation efficiency of the water component is only 23% and this results in a bias of only 25 ms for a true T1 of 1000 ms (2.5% of bias). This simulation does not account for the fat component that can also be excited by the water‐selective pulse if the off‐resonance is too high. In that case, T1 will be a weighted combination of the T1 of water and the excited fat components. However, we only observed off‐resonance frequency above 100 Hz at the interface between the lungs and the liver. The latter having a small fat content, the effect on T1 was negligible (Figure S3). In comparison, the VFA protocol was more sensitive to B0 inhomogeneity and an additional correction factor was necessary to generate accurate water‐specific maps (Figure S2).

B0 inhomogeneity also affects PDFF estimation. High off‐resonance frequency would result in (1) a low effective flip angle, which would be wrongly attributed to a lower content of either component, or (2) in the extreme case, the excitation of water instead of fat or vice versa. Even though PDFF was homogeneous for a large range of off‐resonance frequencies, the PDFF in the liver was not reliable above 75‐Hz off‐resonance (Figure S4). Like with many other methods, Obmann et al. [54] recommends measuring T1 relaxation times in liver segments further away from the lungs. Alternatively, more selective pulses could be used at the expense of a longer TR (Figure S1).

Motion does not result in ghosting artefacts with the current method but might generate blurriness. It might be interesting to improve the effective spatial resolution by implementing motion compensation strategies [55, 56]. Alternatively, a 2D version of the technique could be implemented to reduce the total acquisition time to a single breath hold and hence mitigate the effect of motion. Although the number of slices would be limited, 2D radial MP2RAGE has previously been successfully implemented with six slices interleaved in a single MP2RAGE TR reducing the acquisition time to 9 s [57]. This method could potentially be enhanced by incorporating frequency‐selective pulses, allowing component‐specific T1 estimation and PDFF. Nevertheless, slice‐selective excitation takes longer than hard pulses. Given that the time between two subpulses is constrained, the pulse duration and the gradient duration are limited, hence limiting the slice thickness.

In this work, a free‐breathing method was prioritized to avoid the acquisition time constraints that typically limit scan coverage and resolution. Nevertheless, we demonstrated that we could reduce the acquisition time to maximize patient comfort by sampling less radial spokes and reach an acquisition time of 6 min without undersampling artefacts and significant loss of resolution. Another acceleration strategy would be to lengthen the echo train to 196, as commonly used [58]. This would result in 47% acceleration of the current method (*ETL*_*Eff*_ = 98 versus *ETL_Eff_* = 64 in this study), at the expense of a considerable loss of precision.

As the primary objective of this study was to evaluate the technical feasibility and performance of this new measurement technique, the cohort was intentionally centred on a small, predominantly healthy group to establish a clear baseline. Although the current sample size and the limited number of participants with known pathologies preclude a broad population‐based analysis, these results successfully demonstrate the technique's potential. This foundational work provides the necessary technical validation required before deploying the method in larger‐scale clinical diagnostic studies.

## 5 Conclusion

The MP2RAGE sequence was augmented with radial encoding and frequency selective pulses to generate water‐ and fat‐specific parametric maps of the whole abdomen in a single acquisition under free breathing conditions. This sequence could help in the diagnosis and monitoring of abdominal pathologies involving T1 and/or PDFF changes, especially in noncompliant patients.

## Author Contributions

Nadège Corbin: conceptualization, methodology, investigation, data acquisition, data analysis, writing original draft, review and editing. François Maingault: conceptualization, methodology, investigation, data acquisition, data analysis. Aurélien J. Trotier: conceptualization, methodology, review and editing. Emile Kadalie: resources, data analysis, review and editing. Laurence Dallet: resources. Marc Biran: resources, data acquisition and analysis. Sylvain Miraux: review and editing, funding acquisition. Eric Thiaudière: methodology, investigation, data analysis, review and editing. William Lefrançois: conceptualization, methodology, investigation, supervision, review and editing. Emeline J. Ribot: conceptualization, investigation, data acquisition, data analysis, writing, review and editing, supervision, funding acquisition.

## Funding

This work was supported by the Agence Nationale de la Recherche (ANR‐19‐CE19‐0014). This study was conducted in the framework of the Université de Bordeaux France 2030 programme RRI Impact that received financial support from the French government.

## Conflicts of Interest

The authors declare no conflicts of interest.

## Supporting information


**Figure S1:** Water and fat spectrum obtained from a mixture of pork fat, water and agar (black) with the NMR spectroscopy protocol (STEAM) overlayed on water‐selective (A) and fat‐selective (B) excitation profiles of the binomial pulses with number of sub‐pulses varying from 3 to 7.
**Figure S2:** Water‐selective T1 maps obtained from the VFA protocol (a) without and (b) with correction accounting for B0 inhomogeneity. (c) Normalized difference between T1 maps. (d) Associated B0 map.
**Figure S3:** Water‐selective T1 maps obtained from the MP2RAGE protocol (A) without and (B) with correction accounting for B0 inhomogeneity. (C) Normalized difference between T1 maps. (D) Associated B0 map.
**Figure S4:** Line profiles of the B0 maps (black lines), the corrected (orange lines) and uncorrected (blue lines) PDFF maps in 4 volunteers.
**Figure S5:** Estimation of the inversion efficiency in one slice of the liver of one single participant. Signal after inversion measured across the five ROIs (location shown in yellow) with respect to the signal at the same location on the acquisition without inversion.

## Data Availability

The data that support the findings of this study are available from the corresponding author upon reasonable request.

## Appendix

For radial sampling, all echoes of a GRE train (*n*) have to be accounted for to simulate the signal and subsequently create the LUT. Hence, the equations in Marques et al. [22] have been slightly modified. In addition, the alternation between fat and water selective pulses necessitates the adjustments of the parameters: *TR*_*Eff*_ is the duration between two excitations of the same component; *ETL*_*Eff*_ is the number of pulses within one GRE readout exciting the same component. If we use the same notation as in Marques et al., the signal of one spoke *m* during the GRE1 train can be described as follows:

(A1)
$$\begin{array}{c} \mathrm{GR}E_{\mathrm{TI}1}^{\text{Comp}}\left(m\right)=B_{1}^{-}e^{-\frac{\mathrm{TE}}{T2_{\text{Comp}}^{*}}}M_{0}^{\text{Comp}}\sin\left(\alpha_{1}\right)\\ \left[\left(-\mathrm{eff}.\frac{m_{z,\mathrm{ss}}^{\text{Comp}}}{M_{0}^{\text{Comp}}}\mathrm{EA}+\left(1-\mathrm{EA}\right)\right){\left(\cos\left(\alpha_{1}\right)E_{1}\right)}^{m-1}+\left(1-E_{1}\right)\frac{1-{\left(\cos\left(\alpha_{1}\right)E_{1}\right)}^{m-1}\;}{1-\cos\left(\alpha_{1}\right)E_{1}}\right] \end{array}$$

where *Comp* stands for water or fat; $E1=e^{-TR_{\mathrm{Eff}}/T1_{\text{Comp}}},\mathrm{EA}=e^{-\mathrm{TA}/T1_{\text{Comp}}},\mathrm{EB}=e^{-\mathrm{TB}/T1_{\text{Comp}}}\;\text{and}\;\mathrm{EC}=e^{-\mathrm{TC}/T1_{\text{Comp}}}$; $B_{1}^{-}$ the receive sensitivity; TE the echo time; T2* the effective transverse relaxation time; *M*_0_^*Comp*^ the initial magnetization; *eff* the inversion efficiency; $m_{z,\mathrm{ss}}^{\text{Comp}}$ the longitudinal magnetization of the given component before successive inversion; *TA* the duration between the inversion pulse and the first spoke of *GRE*1; *TB* the duration between last spoke of *GRE*1 and the first spoke of *GRE*2; *TC* the duration between the last spoke of *GRE*2 and the next inversion pulse; and *α*_1_ and *α*_2_ the excitation flip angles used in *GRE*1 and *GRE*2, respectively.

The signal of one spoke *m* during the GRE2 train follows this equation:

(A2)
$$\begin{array}{c} \mathrm{GR}E_{\mathrm{TI}2}^{\text{Comp}}\left(m\right)=B_{1}^{-}e^{-\frac{\mathrm{TE}}{T2_{\text{Comp}}^{*}}}M_{0}^{\text{Comp}}\sin\left(\alpha_{2}\right)\\ \left[\frac{\frac{m_{z,\mathrm{ss}}^{\text{Comp}}}{M_{0}^{\text{Comp}}}-\left(1-\mathrm{EC}\right)}{\mathrm{EC}{\left(\cos\left(\alpha_{2}\right)E_{1}\right)}^{{\mathrm{ETL}}_{\mathrm{Eff}}-m}}-\left(1-E1\right)\frac{{\left(\cos\left(\alpha_{2}\right)E_{1}\right)}^{\mathrm{ET}L_{\mathrm{Eff}}-m}-1}{1-\cos\left(\alpha_{2}\right)E_{1}}\right] \end{array}$$

The resulting signal for each of the two images reduces to a simple average over all echoes:

(A3)
$$S_{\mathrm{TI}1}^{\text{Comp}}=\frac{1}{\mathrm{ET}L_{\mathrm{Eff}}}\sum_{m=1}^{\mathrm{ET}L_{\mathrm{Eff}}}\mathrm{GR}E_{\mathrm{TI}1}\left(m\right)$$

(A4)
$$S_{\mathrm{TI}2}^{\text{Comp}}=\frac{1}{\mathrm{ET}L_{\mathrm{Eff}}}\sum_{m=1}^{\mathrm{ET}L_{\mathrm{Eff}}}\mathrm{GR}E_{\mathrm{TI}2}\left(m\right)$$
