# Supplementary material for: Simultaneous Water‐Specific and Fat‐Specific T1 and Fat Fraction Mapping of the Abdomen in Free Breathing Conditions With a Radially Encoded MP2RAGE
Source: NMR Biomed. 2026 May 8;39:e70297. doi: 10.1002/nbm.70297 (PMC13156445; doi:10.1002/nbm.70297)
Supplement: Supplementary file 1 — Figure S1: Water and fat spectrum obtained from a mixture of pork fat, water and agar (black) with the NMR spectroscopy protocol (STEAM) overlayed on water‐selective (A) and fat‐selective (B) excitation profiles of the binomial pulses with number of sub‐pulses varying from 3 to 7. Figure S2: Water‐selective T1 maps obtained from the VFA protocol (a) without and (b) with correction accounting for B0 inhomogeneity. (c) Normalized difference between T1 maps. (d) Associated B0 map. Figure S3: Water‐selective T1 maps obtained from the MP2RAGE protocol (A) without and (B) with correction accounting for B0 inhomogeneity. (C) Normalized difference between T1 maps. (D) Associated B0 map. Figure S4: Line profiles of the B0 maps (black lines), the corrected (orange lines) and uncorrected (blue lines) PDFF maps in 4 volunteers. Figure S5: Estimation of the inversion efficiency in one slice of the liver of one single participant. Signal after inversion measured across the five ROIs (location shown in yellow) with respect to the signal at the same location on the acquisition without inversion. [file NBM-39-e70297-s001.docx]

**SUPPORTING INFORMATION A**

One limitation of the frequency selective pulse is the sensitivity to B0 inhomogeneity. The lung cavity is particularly challenging for imaging the liver. It affects the MP2RAGE protocol but also the VFA protocol.

A correction of the effective flip angle was therefore implemented to take into account the inhomogeneous B0 field interfering with the frequency selective excitation.

For each voxel $v$, the resulting effective flip angle $\alpha_{eff}$ was computed as follows:

| ${{{\alpha_{eff}(v)=S}_{RF}\left( f(v) \right).C}_{B1}(v).\alpha}_{nom}$ | (S 1) |
| --- | --- |

With $\alpha_{nom}$the nominal flip angle,

$C_{B1}$ is the transmit field efficiency as measured by an additional B1 acquisition

$S_{RF}$ is the theoretical binomial RF pulse frequency spectrum (FigS1)

$f$ is the off-resonance frequency measured with the B0 field map acquisition.

Applying this correction is more demanding in term of computational time because a LUT is required for each voxel as the effective flip angle varies across voxels.

In the case of the Variable Flip Angle method for T1 mapping, the computational time is not affected as transmit field efficiency is always necessary to estimate reliable T1 even without water selective pulse.

The sensitivity to off-resonance frequency and the efficiency of the correction was evaluated for both protocols. We could observe a strong gradient of T1 estimates in the liver with the VFA protocol which was largely compensated with the implemented correction (Fig S2). In this example, the shimming particularly failed.

Despite a larger FOV and therefore a larger shim volume, no gradient was visible in the water-selective T1 maps obtained with the modified MP2RAGE protocol. The correction had negligible impact on the T1 estimates (Fig S3).

The effect of off-resonance frequency was more visible in the PDFF maps at the interface between the lung and the liver where a line profile was analysed (Fig S4). Nevertheless, we could observe that the PDFF was homogeneous across a large range of off-resonance frequencies. It’s only above 75Hz that the PDFF in the liver became unreliable. The effect of the implemented correction had no impact on the estimates either. The robustness to B0 inhomogeneity over a large range of frequency can be explained by the robustness to excitation efficiency of the MP2RAGE method with small flip angles. The inability to correct efficiently at larger off-resonance frequency is due to the fact that, the other component becomes excited and misattributed. Simply correcting the flip angle cannot be the solution. More selective RF pulses, with more sub-pulses (Fig. S1) would provide estimates more robust to B0 inhomogeneity at the cost of a longer TR.

Supporting Figure 1

**
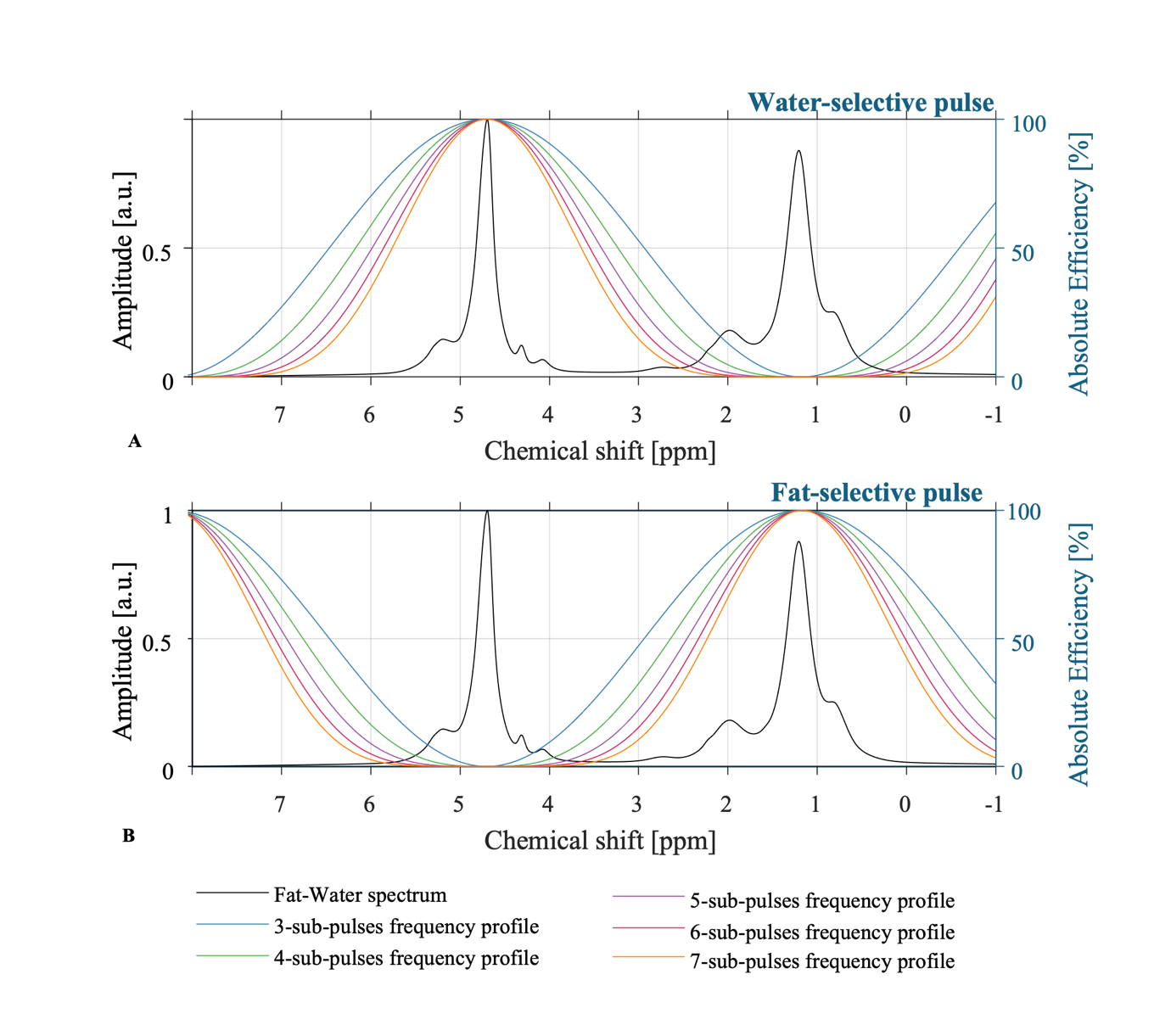
**

**Supporting Information A Figure S1:** Water and fat spectrum obtained from a mixture of pork fat, water and agar (black) with the NMR spectroscopy protocol (STEAM) overlayed on water-selective (A) and fat-selective (B) excitation profiles of the binomial pulses with number of sub-pulses varying from 3 to 7.


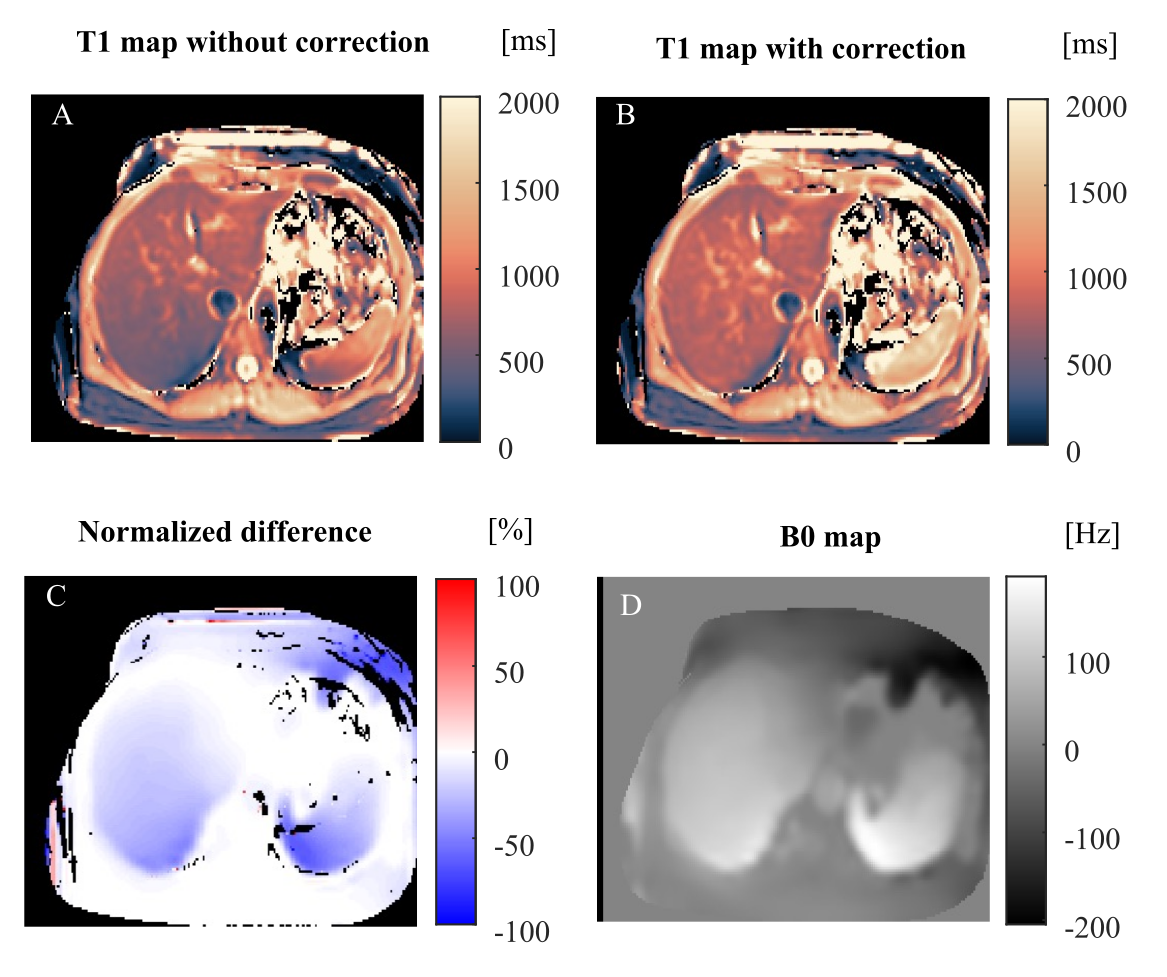


**Supporting Information A Fig. S2:** Water-selective T1 maps obtained from the VFA protocol (a) without and (b) with correction accounting for B0 inhomogeneity. (c) Normalized difference between T1 maps. (d) Associated B0 map.


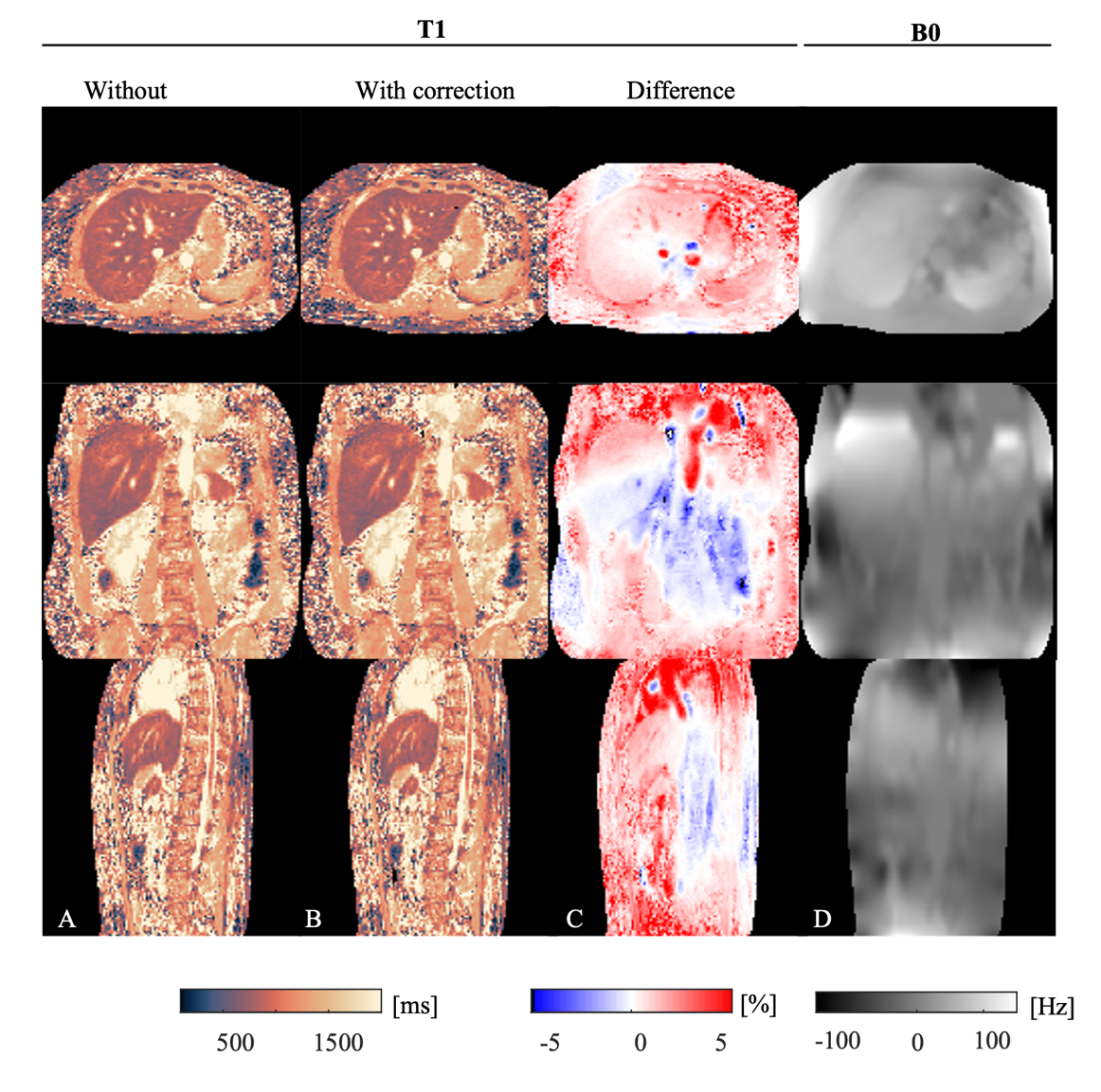


**Supporting Information A Fig. S3:** Water-selective T1 maps obtained from the MP2RAGE protocol (A) without and (B) with correction accounting for B0 inhomogeneity. (C) Normalized difference between T1 maps. (D) Associated B0 map.


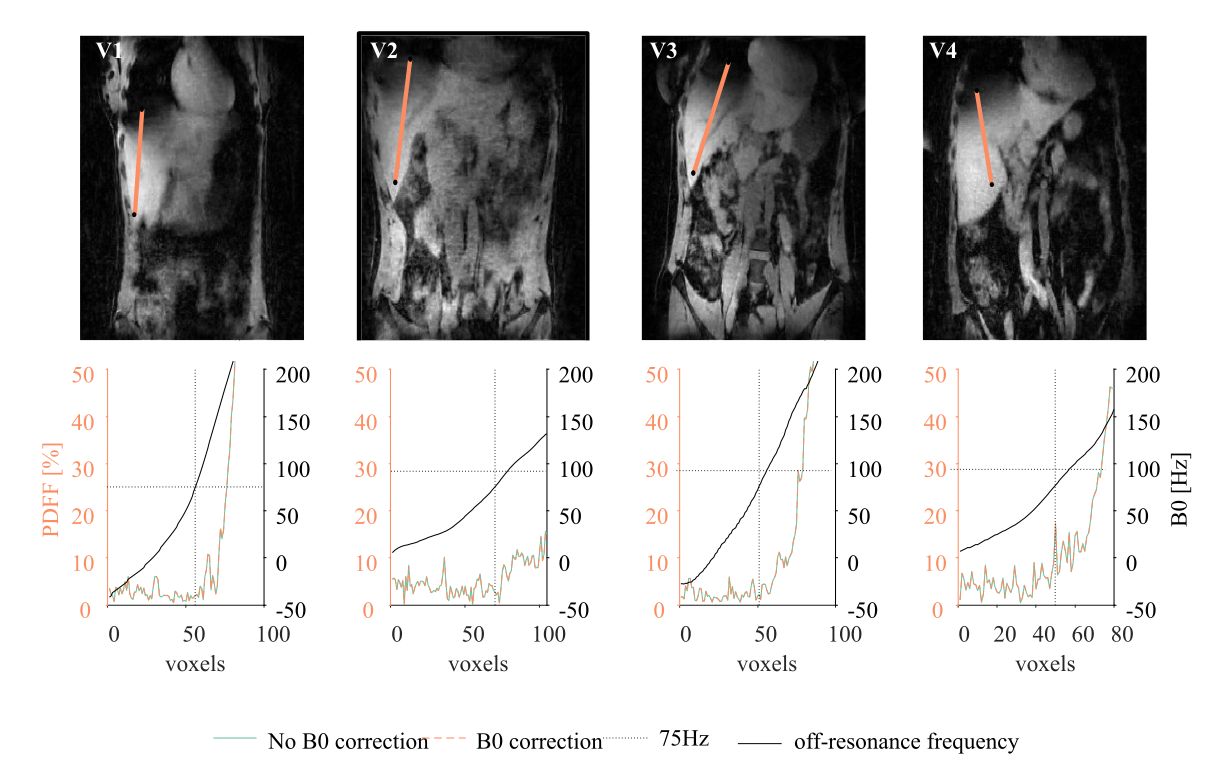


**Supporting Information A Fig. S4:** Line profiles of the B0 maps (black lines), the corrected (orange lines) and uncorrected (blue lines) PDFF maps in 4 volunteers.

**SUPPORTING INFORMATION B**


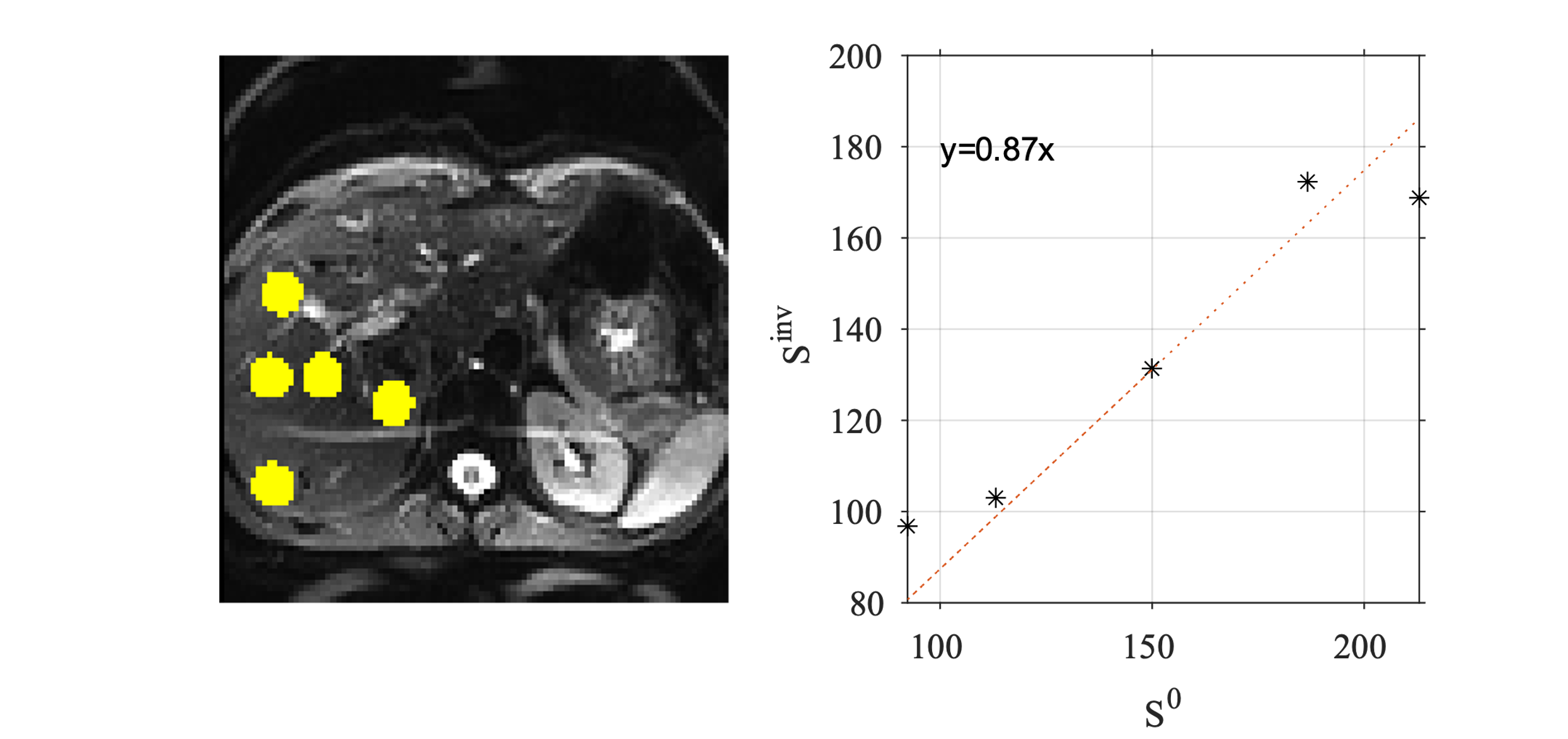


**Supporting Information B Figure S5:** Estimation of the inversion efficiency in one slice of the liver of one single participant. Signal after inversion measured across the five ROIs (location shown in yellow) with respect to the signal at the same location on the acquisition without inversion.
